# Mid-term to long-term outcome and risk factors for failure of 158 hips with two-stage revision for periprosthetic hip joint infection

**DOI:** 10.5194/jbji-10-15-2025

**Published:** 2025-02-11

**Authors:** Moatasem Abuelnour, Conor McNamee, Abdul Basit Rafi, Wolf Hohlbein, Peter Keogh, James Cashman

**Affiliations:** 1 Cappagh National Orthopaedic Hospital, Dublin, Republic of Ireland; 2 School of Medicine, University College Dublin, Dublin, Republic of Ireland; 3 RoMed Klinikum, Rosenheim, Germany; 4 Department of Orthopaedics and Traumatology, Paracelsus Medical University, Salzburg, Austria

## Abstract

**Introduction**: This study aimed to evaluate infection-free survival and outcomes after two-stage revision surgery for hip periprosthetic joint infection (PJI) performed in a specialised arthroplasty unit over 20 years. **Methods**: We retrospectively identified 158 hips (154 patients) treated with two-stage revision surgery for hip PJI between 2001 and 2021. We analysed their data and presented their infection-free survival, re-operation rate, mortality, risk factors and complications. **Results**: The mean follow-up time was 9 (2 to 21.7) years. A total of 22 hips (13.9 %) were re-infected. The infection-free survival was 94.4 % at 2 years, 89.3 % at 5 years, 84.2 % at 10 years, and 82.6 % at 15 and 20 years. The re-operation rate for aseptic causes was 12 %, and the most common cause of re-operation was dislocation (7 %). The cumulative survival for re-operation for aseptic causes was 93.6 % at 2 years, 89.7 % at 5 years, 88.8 % at 10 years, and 82.8 % at 15 and 20 years. The cumulative survival for all-cause re-revision was 88.8 % at 2 years, 80.8 % at 5 years, 74.9 % at 10 years, and 68 % at 15 and 20 years. The mean Western Ontario and McMaster Universities Arthritis Index (WOMAC) hip score significantly improved from 68.3 at the pre-operative stage to 35.9 at 2.1 (2 to 3.3) years, 35.3 at 5.3 (5 to 8.4) years, 38.3 at 11.3 (10–15) years and 43.8 at 18.7 (16.5 to 21.7) years ( p<0.01 ). Duration of antibiotics and gram-negative infection were the only predictive risk factors for re-infection. **Conclusion**: Our results of the two-stage revision protocol for hip PJI were satisfactory and comparable with the best reported outcomes.

## 1 Introduction

Periprosthetic joint infection (PJI) is uncommon (1 %–2 % after primary surgery and 3 %–4 % after revision) (Izakovicova et al., 2019 ) but represents a serious complication. The number of PJIs is increasing due to the rise in joint replacements worldwide, and this has a significant impact on economic and healthcare systems (Dobson and Reed, 2020). Despite the ongoing advances and interests in infection control, there is an increased cumulative revision rate due to infection after primary total hip replacement (THR) (Dale et al., 2012). Several consensus papers have been published for proper diagnosis and treatment of PJI (Parvizi et al., 2018), but still it remains controversial as to which patients might benefit from a single- versus two-stage procedure (Xu et al., 2020).

Nevertheless, the two-stage revision technique is still used by many surgeons since single-stage revision surgery is only recommended for specialised centres and in a group of patients with specific selection criteria (Klouche et al., 2012; Lum et al., 2020). Most of the published studies on two-stage revision have short-term to intermediate-term follow-up since longer follow-up of this group of elderly patients is usually not available due to multiple comorbidities, a high number of deaths and loss of follow-ups (Corona et al., 2020; Kildow et al., 2020).

This study aims to assess intermediate-term to long-term outcomes, the microbiological data and risk factors for failure of two-stage revision surgery for hip PJI in our arthroplasty unit over a period of 20 years. We report our survival outcomes, and we compare them with the published data of other arthroplasty units.

## 2 Patients and methods

From 2001 to 2021, we retrospectively identified cases revised for hip PJI from our hospital records. We included patients with age ≥18 years, who had completed the two stages of hip PJI revision and whose data are available. We excluded patients who had single-stage revision, previous revision for PJI and multiple complex previous revisions for other causes and patients with tumour prostheses. Out of 14 002 primary THRs performed in our unit during this period, there were 1982 hips revised for all causes (14.2 %) and 191 revised for chronic PJI (1.4 %). A total of 158 hips (154 patients) revised for PJI met our inclusion criteria and were treated with the two-stage revision protocol. Most of our patients in this cohort presented with chronic infection ( >4 weeks of symptoms) except seven cases, who had acute presentation (McPherson et al., 2002).

Demographic variables and comorbidities are described in Table 1. The main indication for primary surgery was osteoarthritis in 79.7 % of cases, and the average time from primary THR was 58.2 (1–273) months, while the average time between first and second stages of revision was 15 (2–84) weeks.

Diagnosis and definition of infection depended mainly on a range of clinical, laboratory and radiological tests. This was confirmed by intraoperative signs of PJI specially purulent fluids and implant loosening. No recognised diagnostic criteria were used, but more than one criterion was satisfied in all the cases. Surgical details of the two-stage procedure are described in Table 2. Patients were considered free of infection after the second stage if there was no growth on 5 d culture or only one positive culture with a low virulence or commensal microorganism, a return of serum C-reactive protein (CRP) to normal, and no clinical signs of infection. For the definition of successful clinical outcomes after revision of PJI, the Delphi-based international consensus has been adopted (Diaz-Ledezma et al., 2013). This includes (1) no clinical signs of infection, (2) no subsequent surgical intervention for infection and (3) no occurrence of PJI-related mortality.

**Table 1:** Patients demographics (sample population n=158 ).

Factor	Average/ N	%
Age	65.8 (36–88)	
Sex		
male	107	67.7
female	51	32.3
BMI (kg m^2^)	29.5 (15–44)	
ASA classification		
I–II	104	65.8
III	54	34.2
IV–V	0	
Comorbidities/risk factors		
smoking	33	20.9
alcohol abuse	10	6.3
DM	18	11.4
cardiovascular disease	26	16.5
respiratory disease	18	11.4
rheumatoid/inflammatory	10	6.3
renal/hepatic disorder	16	10.1
malignancy	12	7.6
>1 comorbidity	36	22.8
Indication for primary THR		
OA	126	79.7
RA/inflammatory	10	6.3
posttraumatic	12	7.6
AVN	10	6.3
Time from primary THR (months)	58.2 (1–273)	
Time between first and second stages (weeks)	15 (2–84)	
Cases with >1 primary joint replacement	72	54.6
(Charley class B2)		
bilateral THRs	61	38.6
bilateral THRs + other joint replacement	9	5.7
THR + other joint replacement	2	1.3

BMI, body mass index; ASA, American Society of Anesthesiologists; DM, diabetes mellitus; OA, osteoarthritis; RA, rheumatoid arthritis; AVN, avascular necrosis; THR, total hip replacement.

A total of 129 hips were available for ≥2 years follow-up. The mean follow-up time was 9 (2 to 21.7) years. The average time from second stage of revision surgery to reinfection was 36.1 (2–82) months. The follow-up regime in our unit included phone calls or clinic visits at 6 weeks, 6 months, 2 years, 5 years, 10 years and 15 years. The Western Ontario and McMaster Universities Arthritis Index (WOMAC) score (Bellamy et al., 1988) was collected at different follow-up points by our joint registry team and finally at the end of the study. We combined the follow-up years in intervals with means of 2.1 (2–5 years), 5.3 (5–10 years), 11.3 (10–15 years) and 18.7 (15–20 years). We contacted the patients or their relatives if there was no recent follow-up available on our record system. Patients could have non-routine clinic visit for clinical and radiological assessment of any worrisome signs.

**Table 2:** Operative details of the first and second stages (sample population n=158 ).

Factor		Average/ N	%
Spacer used in first stage			
articulating spacers		116	73.4
	commercial spacer	48	30.4
	femoral stem with cement spacer	34	21.5
	CUMARS procedure	34	21.5
non-articulating cement spacer		39	24.7
no spacer		3	1.9
Implant used in second stage			
fixation type			
	reverse hybrid	52	32.9
	uncemented	39	24.7
	cemented	35	22.1
	hybrid	21	13.3
	not available/no second stage	11	7
femoral stem			
	uncemented long modular stem	78	49.4
	uncemented stem	13	8.2
	cemented long stem	14	8.9
	cemented stem	40	25.3
	proximal femur replacement	2	1.3
	not available/no second stage	11	7
acetabulum cup			
	cemented cup	87	55
	uncemented cup	52	32.9
	dual mobility	6	3.8
	constrained	2	1.3
	acetabulum ring or mesh	4	2.5
	not available/no second stage	11	7
Bone graft		17	10.8
ETO		23	14.6
No second stage		11	7
Surgical approach			
posterior		121	76.6
anterolateral/lateral		25	15.8
transtrochanteric		4	2.5
not available		8	5

CUMARS: custom-made articulating spacer. ETO: extended trochanteric osteotomy.

### Statistical analysis

Data were collected using a standardised electronic form. Continuous variables were summarised with mean and standard deviation and categorical variables with counts and percentages. The Kaplan–Meier estimator was used to calculate cumulative incidences of death, reinfection and reoperation. A multivariate cause-specific Cox proportional hazards regression model was fitted to reinfections. The results of this are reported as hazard ratios with 95 % confidence intervals. WOMAC scores were compared using the Kruskal–Wallis non-parametric test and Dunn's post hoc test to compare between time intervals. All statistical tests were two-sided, and significance was set at alpha = 0.05. Analyses were performed using Python 3.11 (Python Software Foundation) and Prism version 8 (GraphPad software).

## 3 Results

**Figure 1:**
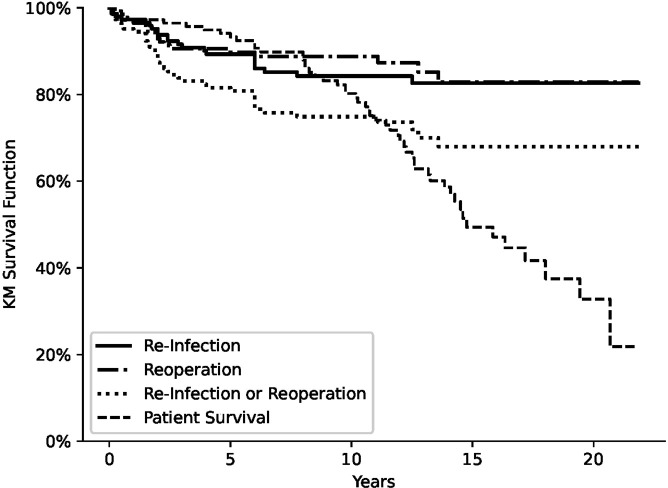
Cumulative incidence of re-infection, re-operation and all-cause re-revision across years of follow-up of two-stage revision hip surgery. Dashed line indicates cumulative incidence of death.

A total of 147 hips completed the treatment, and 22 (13.9 %) hips were identified as re-infected or failed. The infection-free survival was 94.4 % at 2 years, 89.3 % at 5 years, 84.2 % at 10 years, and 82.6 % at 15 and 20 years (Fig. 1). In total, 11 hips did not proceed to the second stage (re-implantation). Analysis of these 11 hips has shown 5 hips did not have a second stage due to multiple comorbidities (ASA grade 3) and they died within an average of 10 (1–17) months, 3 hips did not have surgical data available for second stage, 2 hips had removal of spacer and Girdlestone resection due to severity of infection, and 1 hip had a dynamic spacer in (CUMARS technique, custom-made articulating spacer) (Tsung et al., 2014) and the patient was happy with outcome (WOMAC score 29) at their last follow-up (100 months). The two hips that ended with Girdlestone resection were considered failure and included in survival analysis.

There were 19 hips re-operated on for aseptic causes (12 %) (Table 3). The cumulative survival for re-operation for aseptic causes was 93.6 % at 2 years, 89.7 % at 5 years, 88.8 % at 10 years, and 82.8 % at 15 and 20 years (Fig. 1). Four hips of the re-operated cases had positive bacterial growth from intraoperative samples. In two hips the bacterial growth was considered significant as one hip developed severe infection after re-operation for a loose cup and ended with Girdlestone resection, and the other hip was kept on long-term antibiotics. Both hips were included in failure analysis. Taking the numbers of re-infected and re-operated cases together, the total number of all-cause re-revision is 41 hips (25.9 %). The cumulative survival for all-cause re-revision was 88.8 % at 2 years, 80.8 % at 5 years, 74.9 % at 10 years, and 68 % at 15 and 20 years (Fig. 1). There were three patients with significant radiological and clinical findings, but they were not re-operated on. One patient developed chronic dislocation 3 years after two-stage revision surgery. This patient continued to be treated conservatively due to poor compliance. Additionally, there were two patients with asymptomatic loosening of the femoral stem and acetabular cup respectively.

### 3.1 Functional outcome

At the preoperative stage the average WOMAC score was 68.3±16.2 , and it improved significantly to 35.9±30.7 at the interval of 2.1 (2 to 3.3) years ( p<0.001 ). Thereafter, the score remained relatively constant, 35.3 at 5.3 (5 to 8.4) years, 38.3 at 11.3 (10–15) years and 43.8 at 18.7 (16.5 to 21.7) years ( p<0.01 ), maintaining the improvement from the preoperative stage but not showing further improvements.

**Table 3:** Causes of re-operations other than recurrence of infection (sample population = 158).

Factor	Average/ N	%
Dislocation/instability	11	7
Aseptic loosening of femoral stem	3	1.9
Aseptic loosening of acetabulum cup	2	1.3
PPF	2	1.3
Removal of metals	1	0.6
Total	19	12
Culture-positive intraoperative samples	4	2.5

PPF: periprosthetic fracture.

### 3.2 Mortality

Mortality percentages increased progressively from 5.3 % at 2 years to 68.1 % at 20 years' follow-up after hip two-stage revision surgery. A total of 60 deaths (39 %) occurred by the time of the last follow-up. In the republic of Ireland, the overall mortality rate in 2022 in population aged ≥65 years is 3.8 % ( 29439/782784 ) (CSO, 2023). The mortality analysis included the 11 cases that did not proceed to the second stage; of these, 7 (63.6 %) were dead at the end of the study.

### 3.3 Microbiology

Results of microbiology are summarised in Table 4. There were 53 hips (33.5 %) that were culture-negative; the most common microorganism was *Staphylococcus aureus* (37 hips (23.4 %)) and then coagulase-negative *Staphylococcus* (34 hips (21.5 %)) and *Staphylococcus epidermidis* (31 hips (19.6 %)). Polymicrobial growth was recorded in 37 hips (23.4 %), and MRSA (methicillin-resistant *Staphylococcus aureus*) accounted for 7 hips (4.4 %). None of the culture-negative cases received antibiotics in the period of 2 weeks before the first stage or had autoimmune disease, previous revision for infection or any course of long-term suppressive antibiotics. Only four cases had sinus, and two cases had wash-outs before the first stage.

After the second stage, 17 hips (11.6 %) had culture-positive samples. Samples were considered insignificant if there was only one positive sample, it was a non-virulent microorganism and not the same microorganism isolated from first-stage intra-operative samples. Of these cases, 12 hips (8.2 %) were considered significant and had an extended antibiotic course for further 6–12 weeks. Follow-up and survival analysis of these 17 hips has shown that five hips (29.4 %) got re-infected.

### 3.4 Risk factors

The following risk factors had HR >1.5 but were not statistically significant (Table 5): alcoholism, avascular necrosis (AVN) as indication for primary THR, age >60 years, presence of sinus before first stage, re-implantation with uncemented and hybrid implants, gram-positive organisms (methicillin-sensitive, methicillin-resistant and coagulase-negative staphylococci), and isolation of microorganisms after the second stage. Of our cases, 54.6 % had >1 primary joint replacement (Charley class B2) (Dunbar et al., 2004), but it was not a predictor of worse outcome on the Cox regression analysis as we hypothesised. Samples infected with gram-negative microorganisms (hazard ratio, 24.3 (95 % CI, 2.82 to 208); p<0.01 ) and antibiotic duration >12 weeks (hazard ratio, 3.73 (95 % CI, 1.14 to 12.2); p=0.03 ) were the only predictors of re-infection.

**Table 4:** Microbiology: microorganisms and antibiotics data (sample population n=158 ).

Factor		Average/ N	%
Procedure before first stage			
Aspiration		95	60.1
	culture-positive	41	43.2^a^
	culture-negative	48	50.5^a^
	results not available	6	6.3^a^
	same results as intraoperative samples	54	56.8^a^
Sinus/DAIR		45	28.5
	culture-positive	16	35.6^b^
	culture-negative	3	6.7^b^
	results not available	26	57.8^b^
	same results as intraoperative samples	13	8.3^b^
No procedure/not available		18	11.4
Organism isolated in first stage			
no organism (culture-negative)		53	33.5
*Staphylococcus aureus*		37	23.4
coagulase-negative *Staphylococcus*		34	21.5
*Staphylococcus epidermidis*		31	19.6
polymicrobial (mixed growth)		37	23.4
other gram-positive microorganisms		37	23.4
gram-negative microorganisms		15	9.5
MRSA		7	4.4
candida		2	1.3
anaerobes		1	0.6
Organism isolated in second stage^c^			
from one sample		15	10.2
	*Staphylococcus epidermidis*	5	
	coagulase-negative *Staphylococcus*	3	
	E-coli	2	
	other microorganisms	5	
>1 sample		2	1.4
total		17	11.4
same organism as first stage		7	4.8
Commonly used antibiotics			
vancomycin		94	59.5
rifampicin		62	39.2
ciprofloxacin		59	37.4
flucloxacillin		34	21.5
cefuroxime		26	16.5
fusidic acid		15	9.5
daptomycin		14	8.9
linezolid		10	6.3
ceftriaxone		9	5.7
others (11 antibiotics)		41	25.9
not available		7	4.4
cases received single antibiotic		8	5
Duration of antibiotics			
≤6 weeks		49	31
>6 –12 weeks		42	26.6
>12 weeks		54	23.2
not available		13	8.2

MRSA: methicillin-resistant *Staphylococcus aureus*. ^a^ Population sample n=95 . ^b^ Population sample n=45 . ^c^ Population sample n=147 .

**Table 5:** Potential risk factors for re-infection (sample population n=147 ).

Risk factor	HR	95 % CI	P value
Age			
≤65	1.00		
>65	0.39	0.44–4.42	0.57
BMI			
≤30 kg m^−2^	1.00		
30–40 kg m^−2^	0.86	0.26–2.85	0.71
Sex			
female	1.00		
male	1.10	0.34–3.60	0.87
Smoking			
non-smoker	1.00		
smoker	0.85	0.14–5.08	0.85
Alcoholism ( >10 units a week)			
non-alcoholic	1.0		
alcoholic	4.23	0.59–29.8	0.15
Ischaemic heart disease			
absent	1.0		
present	0.25	0.03–2.26	0.22
DM			
non-diabetic	1.00		
diabetic	0.96	0.13–7.29	0.97
>1 primary joint replacement			
no	1.00		
yes	0.36	0.10–1.29	0.12
ASA			
I–II	1.00		
III	0.90	0.24–3.38	0.88
Indication for primary			
OA	1.00		
rheumatoid/inflammatory	0.21	0.01–3.31	0.27
AVN	5.55	0.68–45.6	0.11
post-traumatic	0.16	0.01–45.6	0.11
Time from first surgery (months)			
<60	1.00		
>60	2.02	0.60–6.86	0.26
Time between the two stages			
≤12 weeks	1.00		
>12 weeks	0.66	0.17–2.52	0.54
Sinus before first stage			
no	1.00		
yes	4.35	0.71–26.53	0.11

**Table 5:** Continued.

Risk factor	HR	95 % CI	P value
Spacer used in first stage			
non-articulating/no spacer	1.00		
articulating	1.25	0.31–5.00	0.75
Implant used in second stage			
cemented	1.00		
uncemented	2.30	0.45–11.8	0.32
hybrid	2.18	0.40–12.0	0.37
reverse hybrid	0.43	0.07–2.54	0.35
Microorganism isolated (first stage)			
no organism (culture-negative)	1.00		
MSSA	3.26	0.68–15.7	0.14
coagulase-negative *Staphylococcus*	2.98	0.70–12.8	0.14
polymicrobial	0.16	0.02–1.54	0.11
other gram-positive	5.05	0.77–33.0	0.09
gram-negative	24.3	2.82–208	<0.01
MRSA	1.89	0.06–58.9	0.72
Microorganism isolated (second stage)			
no	1.00		
yes	1.67	0.39–7.13	0.49
Duration of antibiotics			
≤12	1.00		
>12	3.73	1.14–12.2	0.03

HR: hazard ratio. CI: confidence interval. MSSA: methicillin-sensitive *staphylococcus aureus*.

### 3.5 Complications

The most common surgical complications were dislocation (11.4 %), iatrogenic femur fracture (8.2 %) and repeated first stage (5.7 %) (Table 6). The average time from the second stage to dislocation was 16.7 (1–36) months. A total of 11 hips (7 %) were re-operated on for dislocation, and the rest was stable after closed reduction and non-surgical treatment. For the 13 hips with proximal femur fracture, 7 hips were managed surgically with cables, 3 hips were managed conservatively, and finally one hip was diagnosed 2 weeks later after the second stage and underwent internal fixation with plate and screws. Of the nine hips for which the first stage had been repeated, three hips did not proceed to the second stage due to early death, loss of follow-up and ongoing active infection that ended with Girdlestone hip resection. The other six hips successfully proceeded to second stage.

**Table 6:** Surgical and medical complications (sample population n=158 ).

Factor		Average/ N	%
Surgical complications			
dislocation		18	11.4
	first stage	5	3.2
	second stage	13	8.2
iatrogenic femur fracture		13	8.2
	first stage	3	1.9
	second stage	10	6.3
repeat first stage		9	5.7
post second stage DAIR		1	0.6
skin loss/musculocutaneous flap		1	0.6
foot drop		1	0.6
total		43	27.2
Medical complications			
blood transfusion		59	37.3
urinary retention/UTI		21	13.3
respiratory (LRTI)		18	11.4
cardiovascular		9	5.7
hepatic/renal impairment		9	5.7
bed sores		7	4.4
delirium/confusion		7	4.4
DVT		6	3.8
PE		4	2.5
others		10	6.3
total		150	94.9

UTI: urinary tract infection. LRTI: lower respiratory tract infection. PE: pulmonary embolism. DVT: deep vein thrombosis.

## 4 Discussion

In this series of 158 hips revised with two stages protocol for PJI, survival without re-infection gradually declined until it plateaued, and 84.2 % patients were infection-free at 10 years and 82.6 % at ≥15 years' follow-up. Our results were comparable with best reported outcomes of similar treatment protocols (Petis et al., 2019; Biring et al., 2009; Kunutsor et al., 2015). Hips with previous non-revision interventions for infection like DAIR (debridement, antibiotics, and implant retention) or non-surgical treatment with antibiotics were included, and it did not seem they have worsened our survival outcomes. The reported success rate of two-stage revision is between 65 % to 95 % depending on the length of follow-up and authors' definition of failure (Kildow et al., 2020). Surgeons should be aware of the impact on patients' function, especially between the two stages of revision, increased morbidity and mortality, mental and psychological changes, and general frustration due to uncertainty and fear of recurrence of infection (Walter et al., 2024).

It is estimated that between 17 % and 30 % of patients do not proceed to the second stage during treatment for hip PJI (Gomez et al., 2015). In a series of 162 patients with PJI of hips and knees, Corona et al. (2020) reported an overall rate of eradication of infection of 71.6 %, but this rate increased to 80.6 % when they excluded patients who did not proceed to the second stage. They believed that this group of patients should be considered failure to avoid overestimating the success rate.

We preferred to describe the period without clinical signs of active infection after the second stage of revision as the “infection-free interval” rather than “eradication of infection” as surgeons usually look for serological and clinical signs of absence of active infection to end any invasive intervention; however, dormant infection cannot be excluded on this basis.

The overall mortality in this cohort is better than other studies (20 % at 10 years). Kildow et al. (2022) reported 40.1 % mortality at 5 + years' follow-up, and Wildeman et al. (2021) reported 45 % mortality rate at 10 years' follow-up. These high numbers of mortality may be attributed to the average age >65 years at time of revision and the impact of two-revision protocol on early- and mid-term mortalities.

The preoperative aspiration has shown less accuracy in PJI diagnosis. It is specific but less sensitive in excluding infection (Barker et al., 2021). The results of first-stage intra-operative samples has yielded 33.5 % negative cultures, which is within the range described in other studies (5 % to 42 %, Garabano et al., 2022). The outcome of culture-negative cases in PJI is controversial (Barker et al., 2021); however, only 4 out of 52 culture-negative cases (7.7 %) were documented as re-infection or failure in our cohort. New diagnostic modalities like next-generation sequencing and sonication have been described (Palan et al., 2019). However, surgeons should not expect worse outcomes if no organism is isolated, especially because other authors (Ibrahim et al., 2018; Li et al., 2023) have shown that culture-negative PJI cases have the same or better results compared to culture-positive ones.

Similar to our finding, Tsai et al. (2015) reported *Staphylococcus aureus* as the most common microorganism (29.9 %) in a series of 144 patients. Other studies reported coagulase-negative *Staphylococcus* as the most common microorganism (Petis et al., 2019; Biring et al., 2009). The isolation of *Staphylococcus aureus* was not associated with higher failure rates in our cohort as other studies have shown (Li et al., 2018). Also, polymicrobial infection (23.4 %) was common in our study but was not a risk factor for failure compared to other studies (Kildow et al., 2022). We could not identify any patient in our cohort on long-term suppressive antibiotics, and the current Delphi criteria (Diaz-Ledezma et al., 2013) do not consider patients on long-term suppressive antibiotics as failures.

Isolation of gram-negative microorganism and >12 week duration of antibiotics were predictors of re-infection in our cohort. Other studies described variable risk factors predictive of re-infection, for instance, young age (Bejon et al., 2010), morbid obesity (Houdek et al., 2015), use of chronic antibiotic suppression (Petis et al., 2019), methicillin-resistant staphylococci (Leung et al., 2011), gram-negative infection (Karczewski et al., 2023) and presence of a sinus tract (Xu et al., 2024). The lack of consensus on agreed risk factors is likely due to studies' heterogeneity and small numbers for statistical analysis.

The most common surgical complication was dislocation (8.2 %), which is similar to or less than other studies (Biring et al., 2009; McAlister et al., 2019). Other complications included iatrogenic femur fracture in 13 hips (8.2 %). Only two hips (1.3 %) were revised for postoperative periprosthetic femur fracture, which is significantly better than other similar reports (Petis et al., 2019). Surprisingly, only one case operated on by posterior approach had a foot drop due to sciatic nerve injury (0.6 %); this is similar to the risk of nerve injury after primary THR.

Other techniques have been developed like one-stage revision of PJI, with less complications, better function and similar outcomes (Goud et al., 2023). In a cost analysis study, Klouche et al. (2010) reported 70 % more cost with two-stage revision surgery. Nace et al. (2023) described a 1.5-stage revision in which a cement impregnated with 10 % antibiotics was used to fix the cemented components. No re-implantation is intended until aseptic failure of the first-stage implant. In our cohort, 21.5 % were treated similarly with the CUMARS procedure (Tsung et al., 2014); however all of them except one proceeded to second-stage revision in a short period.

### Study limitations

This a single-centre retrospective study and inherently contains biases and lack of control or standardisation of different variables and comorbidities. Currently the most accepted and validated criteria for PJI diagnosis are the Musculoskeletal Infection Society (MSIS) criteria (Parvizi et al., 2018), but they were not applied to our retrospective cohort. The results of preoperative hip aspiration were only available in 60 % of our cases, and we did not have enough data for synovial cell counts. Our results could be overestimated due to the influence of different factors. The diagnosis was mainly based on surgeons' experience with clinical and serological confirmation; however, we can not exclude the possibility that non-infected hips have been revised with the two-stage protocol. The study retrospectively spanned >20 years, where different techniques, implants and antibiotic protocols have been introduced over this time. The average time between the two stages of revision was 15 weeks (2–84), which is longer than recommended. However, we are not aware of any study that has discussed the effect of time interval between the two stages of hip PJI revision on survival outcomes. Most cases that did not proceed to the second stage were not included in our analysis of failures. However, the number of cases without re-implantation was relatively small (11 cases (7 %)), compared to other studies that reported an average of 19 % of cases without re-implantation (Bourgonjen et al., 2021).

## 5 Conclusion

Our results for treatment of chronic hip PJI using two-stage revision protocol were similar to the best reported outcomes using the same protocol. Surgeons and patients should be aware of the complexity of hip PJI, the high mortality rate and controversy on what is successful for either the surgeon or the patient.

## Data Availability

The data presented in this study are available upon request from the corresponding author. The data are not publicly available due to the privacy concerns regarding protected health information.
